# An Ototoxicity Grading System Within a Mobile App (OtoCalc) for a Resource-Limited Setting to Guide Grading and Management of Drug-Induced Hearing Loss in Patients With Drug-Resistant Tuberculosis: Prospective, Cross-Sectional Case Series

**DOI:** 10.2196/14036

**Published:** 2020-01-14

**Authors:** Cara Hollander, Karin Joubert, Natalie Schellack

**Affiliations:** 1 School of Pharmacy Sefako Makgatho Health Sciences University Ga-Rankuwa South Africa; 2 Department of Speech Pathology and Audiology University of the Witwatersrand Johannesburg South Africa

**Keywords:** drug-resistant tuberculosis, ototoxicity, grading system, eHealth, OtoCalc

## Abstract

**Background:**

Tuberculosis (TB) affects millions of people worldwide and is treated with medication including aminoglycosides and polypeptides. Individuals respond differently to medications as a result of their genetic inheritance. These differences in genetic inheritance can result in the underdosing or overdosing of medication, which may affect the efficacy or, in the case of aminoglycosides and polypeptides used in the treatment of all forms of TB, result in ototoxicity. When ototoxicity is detected, physicians should adjust dosages to minimize further ototoxicity and hearing loss; however, there are no suitable grading systems to define significant hearing loss.

**Objective:**

The aim of this study was to develop a standardized grading system by making use of an electronic health (eHealth) platform to ensure that a user-friendly method was available to interpret hearing test results, calculate significant hearing loss, and provide recommendations with regard to dosage adjustments and management. It further aimed to establish the sensitivity of the newly developed grading scale.

**Methods:**

This grading system was developed in South Africa based on data that were obtained from an audiology and pharmacokinetic study on patients with drug-resistant TB (DR-TB) at two DR-TB units at state-run hospitals. This feasibility study employed a prospective, cross-sectional, exploratory, descriptive case series research design, with a total of 22 participants. Participants underwent audiological and pharmacological assessments at baseline and every 2 weeks for the first 3 months of treatment. Various professionals (8 in total) were subsequently involved in the development of the eHealth system, including a software engineer, four audiologists, a pharmacist, a medical doctor, and a nurse. The app underwent 14 modifications that involved aspects of data storage, ease of usability, grades, and the risk factor checklist.

**Results:**

An ototoxicity grading system within a mobile app for use by doctors, nurses, and audiologists was developed for patients with DR-TB. The purpose of this user-friendly ototoxicity calculator, *OtoCalc*, is to (1) assist health professionals in assessing patients for ototoxicity, (2) establish the clinical significance of ototoxicity by calculating the grade of hearing loss, (3) monitor the progression of hearing loss, and (4) enable systematic referral and management of patients according to their needs.

**Conclusions:**

This newly developed system is more sensitive than the existing grading methods for determining ototoxicity in patients with DR-TB. This app needs to be trialed in a larger sample to establish data security, ease of use, and suitability within this population.

## Introduction

### Background

Tuberculosis (TB) is a contagious, potentially lethal airborne disease [1]. The overall burden of TB has been declining at an annual average of 2% [2], yet the number of patients with drug-resistant TB (DR-TB) is increasing [3]. In 2016, there were an estimated 600,000 new multidrug-resistant and rifampicin-resistant TB cases [2].

Aminoglycosides, such as kanamycin, and some polypeptides, such as capreomycin, are used in the treatment of DR-TB. Although the guidelines are being re-evaluated and many less toxic drugs have been introduced [4], aminoglycosides are still used in countries that do not have access to these more expensive drugs [5]. Aminoglycosides are inhibitors of prokaryotic protein synthesis at commonly accepted therapeutic concentrations. However, they may also affect the protein synthesis of cells at larger concentrations, leading to toxicity such as ototoxicity, vestibulotoxicity, and nephrotoxicity [6]. The toxicity profile of capreomycin is similar to that of aminoglycosides [7].

The prevalence of cochlear damage because of aminoglycosides ranges from 7% to 90% [7]. This variability could be because of a potential underreporting and the lack of distinct parameters for examining ototoxicity [7,8]. Different studies also detail different dosages and methods of monitoring hearing loss, which could account for the different results in terms of incidence [9].

Monitoring and management of ototoxicity are concerns [10], and currently, no comprehensive protocols for monitoring ototoxicity in this population exist; different methods are used worldwide. Interdisciplinary communication with monitoring and management is rare. Monitoring allows for intervention, which permits the patients and their families to maintain effective communication, should the hearing loss worsen, in cases where alternative treatment may not be a suitable or viable option [11]. Hence, there is a need for a detailed and descriptive monitoring tool to guide this interdisciplinary input, specifically in the DR-TB population.

A grading system provides a systematic framework to classify the loss of hearing from baseline. It allows a clear understanding of the degree of this loss and assists with methodical and uniform management. However, existing ototoxicity grading systems, namely, the National Cancer Institute’s Common Terminology Criteria for Adverse Events (CTCAE) ototoxicity grades [12], the grading system by Theunissen and colleagues (TUNE) [13], Brock Hearing Loss Grades [14], the Chang and Chinosornvatana grading scale [15], and the International Society of Pediatric Oncology Boston Ototoxicity Scale [16], do not seem relevant, as they were not developed for this DR-TB population and frequencies between 12.5 kHz and 20 kHz. Furthermore, the interpretation of audiological results seems to differ among individuals. This difference makes the management of the DR-TB difficult for physicians, as there are various opinions of a *significant change* in hearing to motivate a change in the drug regimen of these patients. There are also no grading scales that correspond to management recommendations, where audiologists may not be present for the skilled interpretation of the grades. There has been a need identified for ototoxicity grading systems to allow clinicians to understand the severity of ototoxicity and standardized treatment based on the grades [7]. In addition, it is unclear as to which frequencies tested actually dictate clinical hearing loss. Textboxes 1 and 2 describe the existing grading scales in adult and pediatric populations, respectively, but these are not specifically developed for this DR-TB population.

It is generally the audiologists’ role to interpret audiograms; however, audiologists are often not available at all TB management clinics in developing countries. If a trained nurse conducts audiological testing, a grading system will assist in the interpretation and guide the management protocol. Ototoxicity monitoring can only be successful when a fixed regimen is followed and involves numerous health professionals (oncologists, ear-nose-throat specialists, audiologists, nurses, and clinical pharmacists) as well as the patients [17].

**Existing ototoxicity grading scales according to National Cancer Institute’s Common Terminology Criteria for Adverse Events and TUNE for adults.**

National Cancer Institute’s Common Terminology Criteria for Adverse Events (grades up to 8 kHz) [11,12]

- Originally developed for children yet has adult applications
- Grade 1: Threshold shift or loss of 15-25 dB relative to baseline, averaged at two or more adjacent frequencies in at least one ear
- Grade 2: Threshold shift or loss of >25-90 dB, averaged at two adjacent test frequencies in at least one ear
- Grade 3: Hearing loss sufficient to indicate therapeutic intervention, including hearing aids. Adults: >25-90 dB, averaged at three adjacent test frequencies in at least one ear
- Grade 4: Indication for cochlear implant and requiring additional speech–language-related services. For adults, a profound hearing loss is a*t* >90 dB HL

TUNE by Theunissen and colleagues (grades up to 12.5 kHz) [13]

- Developed for adults
- Grade 0: No hearing loss
- Grade 1a: Threshold shift ≥10 dB at (8-10-12.5) or subjective complaints in the absence of a threshold shift
- Grade 1b: Threshold shift ≥10 dB at (1-2-4)
- Grade 2a: Threshold shift ≥20 dB at (8-10-12.5)
- Grade 2b: Threshold shift ≥20 dB at (1-2-4)
- Grade 3: Hearing level ≥35 dB HL at (1-2-4) de novo
- Grade 4: Hearing level ≥70 dB HL at (1-2-4) de novo

**Existing ototoxicity grading scales for children.**

Brock (1991; grades up to 8 kHz) [14]

- On the basis of absolute hearing level rather than change from baseline and bilateral loss
- Grade 0: <40 dB at all frequencies
- Grade 1: ≥40 dB at 8 kHz only
- Grade 2: ≥40 dB at 4 kHz and above
- Grade 3: ≥40 dB at 2 kHz and above
- Grade 4: ≥40 dB at 1 kHz and above

Scale by Chang and Chinosornvatana (grades up to 12 kHz) [15]

- On the basis of absolute hearing levels and a modification to the Brock scale. It detects milder degrees of hearing loss rather than change from baseline and is based on bilateral loss
- Grade 0: ≤20 dB at 1.2 and 4 kHz
- Grade 1a: ≥40 dB at any frequency 6-12 kHz
- Grade 1b: >20 dB and <40 dB at 4 kHz
- Grade 2a: ≥40 dB at 4 kHz and above
- Grade 2b: >20 dB and <40 dB at any frequency below 4 kHz
- Grade 3: ≥40 dB at 2 or 3 kHz and above
- Grade 4: ≥40 dB at 1 kHz and above

### Objectives

The mobile health (mHealth) trend, which uses mobile devices and associated technology for health interventions, provides an unprecedented opportunity to transform health services available to people across the globe, specifically in developing countries where the public health system is often described as dysfunctional [18]. mHealth care can become widely available and assist health care at the regional, community, and individual levels [19]. The aim of this study was to develop a standardized grading system by using an electronic health (eHealth) system to ensure a user-friendly method was available to interpret hearing test results, calculate significant hearing loss, and provide recommendations with regard to dosage adjustments and management. It further aimed to establish the sensitivity of the newly developed grading scale.

## Methods

### App Design and Development From an Audiology and Pharmacokinetic Study

A mobile app was developed as part of a project conducted in 2016 in South Africa. This mobile app was based on data that were obtained from an audiology and pharmacokinetic study on patients with DR-TB at two DR-TB units at state-run hospitals. The study was a joint study among the Audiology Department at the University for the Witwatersrand, Division of Clinical Pharmacy at Sefako Makgatho Health Sciences University, Wits Health Consortium Clinical HIV Research Unit, and the South African Medical Research Council. The medical ethics boards of the University of the Witwatersrand and various hospitals approved this study.

The intention of this app was to propose a standardized grading system; enhance the utilization of eHealth; and develop a user-friendly mobile app to interpret hearing test results, calculate significant hearing loss, and provide recommendations with regard to dosage adjustments and management for adult patients with DR-TB. The app was designed to streamline the protocol for testing adult patients treated with aminoglycosides for DR-TB and to assist with a uniform interpretation of a significant hearing loss and consequently the collection of reliable statistics based on a similar method of collection and grading of the hearing loss. Furthermore, its aim was to assist a variety of health care professionals who are not necessarily trained in audiology, to guide with recommendations and counseling, particularly when audiologists are not available in poor-resource contexts.

Data from a feasibility study that employed a prospective, cross-sectional, exploratory, descriptive case series research design were used in the development of the app. A total of 22 participants participated in this multisite study at Helen Joseph and South Rand Hospitals (two of the three main hospital-based TB focal points that treat patients with DR-TB in the Johannesburg area of the Gauteng province in South Africa). This study used purposive sampling, a nonprobability sampling strategy to select participants [20], whereby specified inclusion and exclusion criteria were determined. Inclusion criteria included males and females; HIV-positive and HIV-negative patients; those who anticipated treatment with an injectable (either kanamycin or capreomycin) for at least 3 months; those who had proficiency in English, isiZulu, and isiSotho (to ensure study procedures could be performed accurately); those who were aged between 18 and 55 years to exclude presbycusis [21]; those who had normal middle ear status at baseline, as determined by otoscopy and tympanometry; and those who had hearing loss no greater than 70 dB at three or more frequencies bilaterally. The study excluded patients with diabetes mellitus [22] and a history of significant substance abuse.

The sample size was initially calculated as 60 based on a power calculation. However, 12 months after the initiation of recruitment, enrollment ceased as saturation (after adequate enrollment for a feasibility study) was reached. Only 22 participants were enrolled owing to various reasons such as refusal to participate, being transferred elsewhere, and not fitting within the inclusion/exclusion criteria. Although this study had a small sample size, it is essential to place the sample size in the broader context of TB treatment adherence, where adherence is often poor [23].

Data collection included several hospital standard-of-care procedures such as weight, height, HIV status, creatinine level, CD4 count, potassium level, thyroid-stimulating hormone level, liver enzyme levels, and a full blood count. Study-related procedures included a detailed case history as well as audiological and pharmacological assessments at baseline and every 2 weeks for the first 3 months of treatment. Audiological procedures included otoscopy [24], tympanometry [25], pure-tone air-conduction audiometry (up to 16 kHz), and distortion product otoacoustic emissions (up to 12 kHz) [25]. Pharmacological measures included kanamycin or capreomycin elimination rate (ke) and half-life (t_1/2_) from peak and trough concentrations (C_max_ and C_min_, respectively), volume of distribution (Vd), and calculation of the creatinine clearance (CrCl). Finally, the overall outcome of treatment was evaluated in relation to the pharmacokinetics.

From observation and individual consultation with medical doctors in the field, through this feasibility study, many are unable to interpret audiograms when provided. Hearing loss guides the motivation for changes in the treatment regimen. These changes in the treatment regimen may involve the doctor changing the drug and, perhaps, reducing the days of therapy. Thus, it is essential that the doctors and nurses understand the correct interpretation of audiograms to implement these changes and counsel the patients.

Although audiologists generally interpret audiograms, they are often not employed at TB management clinics. If a trained nurse, as recommended above, conducts audiological testing, a grading system will assist in the interpretation. This will guide the management protocol.

In addition, many audiologists are unsure of how to classify a significant ototoxic hearing loss and when to advise the medical doctor to adjust the medication regimen.

### Need for Mobile App Development

The intention of this app was to propose a standardized grading system; enhance the utilization of eHealth; and develop a user-friendly mobile app to interpret hearing test results, calculate significant hearing loss, and provide recommendations with regard to dosage adjustments and management for adult patients with DR-TB. Patients in poor countries generally have less access to health services than those in more developed countries. Key solutions to assist these people can involve innovations in the delivery of services and the regulation of health services. The use of eHealth can assist the vulnerable population in accessing the required services [26]. Thus, this app was designed to streamline the protocol for testing adult patients treated with aminoglycosides for DR-TB and to assist with a uniform interpretation of a significant hearing loss, and thereby the collection of reliable statistics based on a similar method of collection and grading of the hearing loss. The study aimed to assist a variety of health care professionals who are not necessarily trained in audiology, to guide with recommendations and counseling, particularly when audiologists are not available in resource-poor contexts.

### Mobile App Development and Accessibility

Data from the audiology and pharmacokinetic study were analyzed and yielded information regarding high and ultrahigh frequency hearing loss within the first 3 months of treatment. Hearing loss did not extend to the lower frequencies, which is likely because of the short duration of data collection. However, various cultural, social, and context-specific issues need to be considered, including the acknowledgment of patients defaulting on their treatment, the influence of African traditional healers on many patients, the 11 official languages spoken in South Africa that can contribute to communication difficulties, and the limited number of audiologists available in South Africa to implement and manage an ototoxicity monitoring protocol.

The app was primarily developed by a software engineer and piloted by various health professionals. Data security was specifically considered. The mobile app allows for patient data entry directly into the app via any mobile device connected to the Web. Despite the capability for data transmission over wireless networks, we elected only to allow users involved in the field of TB to participate. Collected data were stored on the mobile devices in field and in a password-protected cloud, where only two users (developers) had access. All users’ phones required to be password protected, and the passwords needed to be entered upon opening the app. Patient-specific data could only be obtained with the patient’s identity and hospital numbers to ensure only authorized personnel had access. Both Android and iOS operating systems were selected to reach as many users as possible.

The data backups comprised both active and manual backups. Both the active backup and archives used the same encryption as the database. To minimize the risk of data mitigation failure, the data were stored in different geographic locations. The infrastructure was powered by a small Google Cloud. All access to the data can be logged and time stamped, and a log file can be provided in unlikely cases. The raw data can be exported in .csv, .txt, and .json formats to Microsoft Excel for data analysis and statistical purposes.

The cost of the app was equal to the data costs involved in the original download of the app. The developers tried to ensure Wi-Fi availability where possible to reduce these charges.

### Process Flow for the App-Based Data Collection Tool

Data collection via the app involved a user using the app as either a one-off user (ie, as an ototoxicity calculator) or a continued user, whereby he or she would require permission to *reaccess* patient data for more than a one-off use. Once the user had signed up as a continued user, their application was vetted and access was either denied or granted.

**Figure 1:**
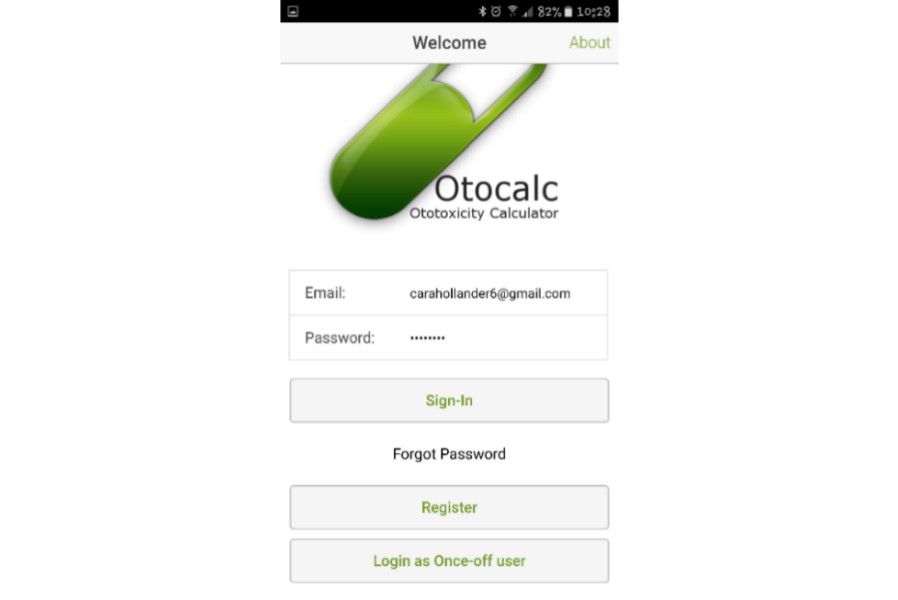
*OtoCalc* home page.

**Figure 2:**
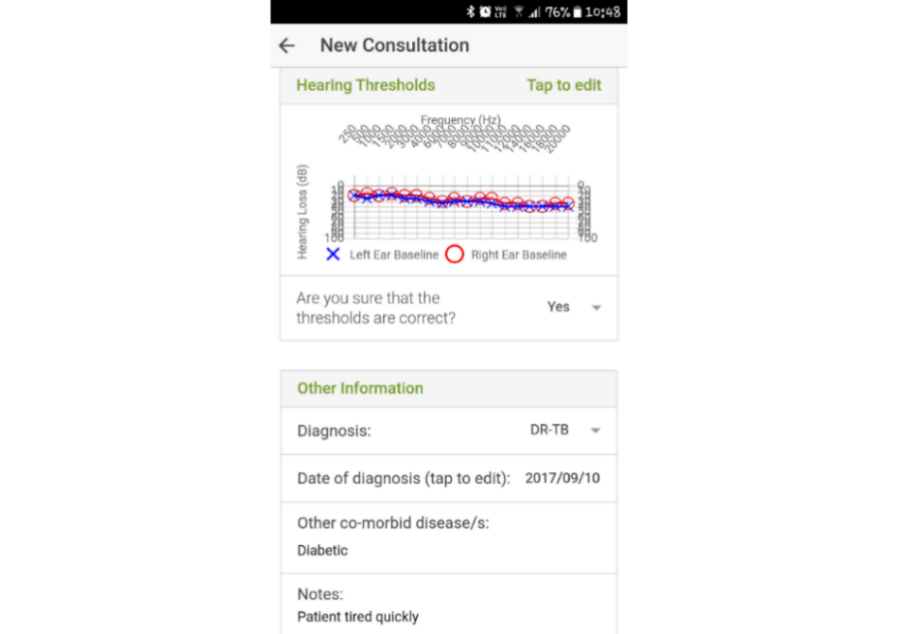
Screenshot depicting the new consultation page.

**Figure 3:**
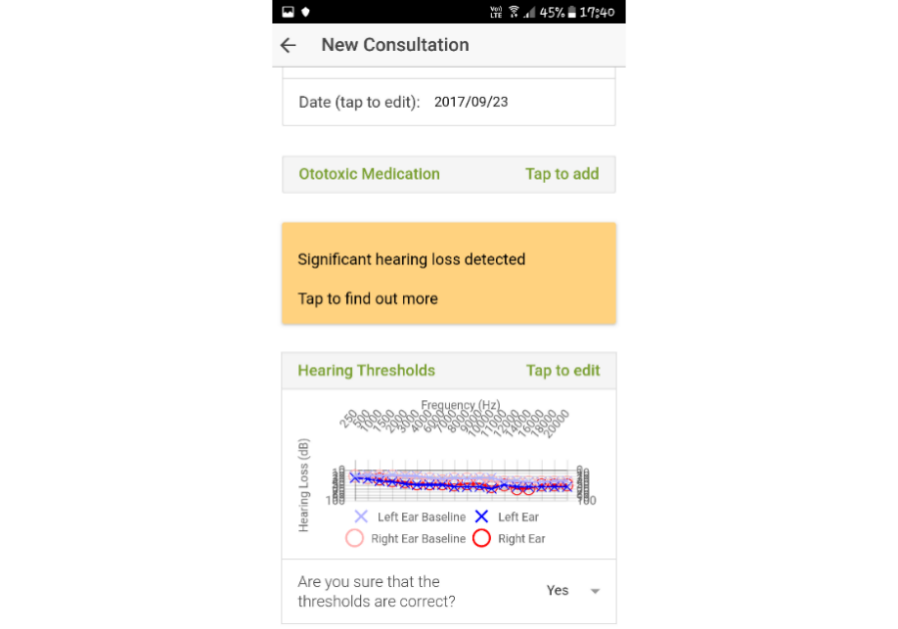
Screenshot of the page when follow-up consultation has been entered and results are calculated.

Subsequently, they could begin using the app by selecting *New Patient*, whereby they would enter basic identifying data, data on more in-depth variables such as weight and medication, and then a risk factor checklist. Baseline audiometric data could be entered, following which the session could be saved. Upon a subsequent patient visit, new data could be updated and new audiometric results could be entered. Upon entering a follow-up audiogram, the app automatically calculated the *grade* of hearing loss and provided explanations and recommendations. These explanations can be shown to the patient, with a management plan. Figures 1-3 display a few screenshots of the various stages of *OtoCalc*.

**Figure 4:**
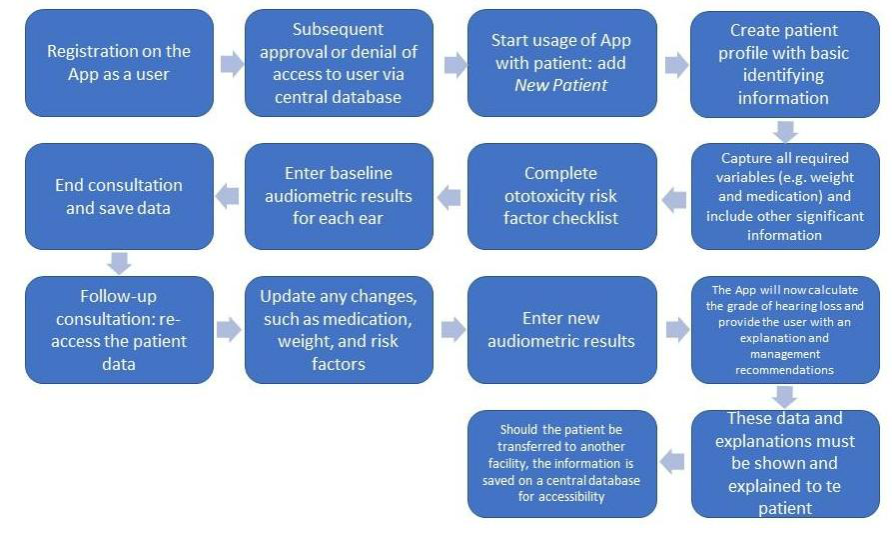
Process for the app-based data collection tool.

All results are stored in a central database; hence, if the patient were to be transferred to another treatment facility, their results and management plans could be accessed and continued. Figure 4 illustrates this process. 

### Usability and Piloting of the App

Various professionals were involved in the piloting of the app, namely, a software engineer, four audiologists, a pharmacist, a medical doctor, and a nurse.

Each professional was asked to evaluate seven aspects on a scale from 1 to 10 (10 is the highest score). When there was one or more ratings of ≤7, the app was modified by the developer. The aspects evaluated were (1) ease of use, (2) ability to reaccess the data for follow-up consultations, (3) coverage of relevant aspects of the app (ie, risk factors), (4) accessibility in terms of usage when there is no internet connection, (5) ability to follow through with the recommendations as detailed in the app, (6) ease of implementing a variety of test protocols within the app (not all frequencies are always tested at various sites), and (7) relevance of the grading scale to the DR-TB population.

The changes required included multiple user accessibility modifications and changes with the *recommendations* framework as well as additions and deletions of aspects such as the risk identification checklist, storage of the data on the database, and grading descriptions. From initial development, the app underwent 14 modifications until it was ready for upload on the Android and iOS stores for download.

All eight professionals and data collectors were confident in using the app and found the tool easy to use. Most of the data collectors found the functions of the app to be well integrated and consistent. All eight data collectors agreed that the app could be useful in future ototoxicity management.

### Validation of the App

This newly developed app needs to be trialed and further piloted by various health care professionals, such as doctors, nurses, pharmacists, and audiologists, within the DR-TB population.

## Results

### OtoCalc Description

The app allowed two-fold usage. It was predominantly used as an *ototoxicity calculator* to assist the health professional in assessing the patient for ototoxicity and establishing the clinical significance of this ototoxicity. This can be used for a *one-off* calculation, and the patient details are not stored on the system. The user does not need to register for this option. The second use of the app was as an ototoxicity monitoring app that would be available to those working within the field of TB, specifically DR-TB management. It can be used for ongoing care by the health care professional who will update the variable component of the app at each visit to monitor the progression of hearing loss. This system provides a systematic basis for referral and management of patients according to their needs.

In total, the grading system comprises eight grades, with each grade containing specific information regarding the description of the class (Multimedia Appendix 1). This entails the decibels, specific frequencies, and whether it would be unilateral or bilateral. The description of this grade is provided to the *layperson*, describing the clinical difficulty that a patient would experience. This would involve explanations of whether the patient will likely notice a deterioration in hearing; whether the speech sounds are affected; the clinical symptoms the patients would experience in everyday life, for example, as a result of the loss of specific frequencies; and whether the current drug regimen may cause progression in speech frequencies when it has not already. It also details when the patients may start to experience social and emotional difficulties as a result of deterioration in hearing.

The mobile app then correlates the grade to management recommendations for the multidisciplinary team, including the medical doctor, nurse, and audiologist. This would include dosage adjustments or motivation for dosage adjustments; the use of an alternate drug or motivation for alternate drugs; appropriate and relevant referrals for the medical doctors, nurses, and audiologists; and audiological recommendations including diagnostic assessments, implementation of communication strategies, and amplification.

Finally, for each grade, counseling recommendations are listed for the treating team to cover. This is specifically important for the patient and family to understand hearing loss as well as its possible management in the future. It is important for patients to not only understand that DR-TB treatment is imperative, despite the hearing loss, but also to know that they are not alone and management is possible. The aspects to cover are listed as points, if the medical or allied professional is unskilled and not knowledgeable in this field (Multimedia Appendix 1).

Grades 1 and 2 involve frequencies in the range of 9-20 kHz. As ultrahigh frequencies (above 8 kHz) are often not conducted, most individuals present with either a grade 3 and above or hearing that is not gradable (no ototoxicity). When the hearing presents as grade 3, the loss in hearing is likely interfering with daily functioning, as it involves the speech frequencies (Multimedia Appendix 1).

### Sensitivity of OtoCalc

The data from the feasibility study were used to establish the sensitivity of *OtoCalc*. The sensitivity of *OtoCalc* was compared with the existing grading/classification systems, namely, TUNE, CTCAE, and the American Speech-Language-Hearing Association (ASHA) system (Table 1). When using *OtoCalc*, ototoxicity was identified in all but two ears (participants 2 and 4). This is more than the number for the other scales, namely, TUNE and CTCAE, as well as the ASHA system. Table 1 exhibits the sensitivity of the *OtoCalc* scale and its ability to identify a change in hearing more often than the other scales. The specific grades further depict changes in certain frequencies; for example, grade 3 indicates a loss in the high frequencies and not ultrahigh frequencies, whereas grades 1 and 2 indicate ultrahigh frequency hearing loss. The results for patients 3, 11, and 13 were not included, as only a baseline assessment was conducted for them.

**Table 1:** Sensitivity of *OtoCalc* (N=15).

Patient	Weeks in the study and ear		Grade according to different scales				Presence of ototoxicity
			*OtoCalc*	TUNE^a^	CTCAE^b^	ASHA^c^	
1	4	Right	2	2a	N/A^d^	Yes	
		Left	2	1a	N/A	Yes	
2	2	Right	2	0	N/A	No	
		Left	0	1a	N/A	No	
4	12	Right	3	1a	N/A	Yes	
		Left	0	0	N/A	No	
5	12	Right	1	1a	N/A	Yes	
		Left	3	2a	N/A	Yes	
6	4	Right	1	0	N/A	Yes	
		Left	2	0	N/A	Yes	
7	12	Right	2	2a	N/A	Yes	
		Left	1	0	N/A	Yes	
8	12	Right	2	2a	N/A	Yes	
		Left	1	1a	N/A	Yes	
9	12	Right	3	1a	N/A	Yes	
		Left	1	1a	N/A	Yes	
10	12	Right	3	2a	N/A	Yes	
		Left	1	2a	N/A	Yes	
12	2	Right	3	2a	1	Yes	
		Left	1	1a	N/A	Yes	
14	8	Right	1	1a	N/A	Yes	
		Left	3	1a	N/A	Yes	
15	2	Right	3	1a	N/A	Yes	
		Left	4	1a	N/A	Yes	
16	8	Right	1	2a	N/A	Yes	
		Left	3	1a	N/A	Yes	
17	8	Right	5	2a	N/A	Yes	
		Left	3	1a	N/A	Yes	
18	10	Right	1	1a	N/A	Yes	
		Left	2	2a	N/A	Yes	

^a^TUNE: Theunissen and colleagues.

^b^CTCAE: Common Terminology Criteria for Adverse Events.

^c^ASHA: American Speech-Language-Hearing Association.

^d^N/A: not applicable.

## Discussion

### Principal Findings

This study led to the development of *OtoCalc*, a detailed and sensitive ototoxicity grading system specifically to be used as a mobile app to assist in hearing loss grading and management resulting from the treatment for DR-TB.

Hearing loss can affect one’s quality of life by affecting the ability to communicate. This hindered or lack of communication can affect socialization and professional opportunities.

Hearing loss can also further result in emotional and social difficulties, including loneliness, decreased self-esteem, and various other difficulties [27]. This can impact an individual’s ability to function and obtain work. Severe depression can become a comorbid disability resulting in the need for further disability grants, again placing financial burden on the country. An individual’s mental state also impacts his/her family and their entire unit’s well-being.

A robust ototoxicity-monitoring protocol requires synergistic relationships among all the involved health care professionals, including positive patient-clinician relationships [17]. This mobile app includes a team approach that can foster patient-centered/family-centered rehabilitation. Improvement in the understanding of the cellular and molecular underpinnings of ototoxicity is critical to developing individualized preventive and rehabilitation strategies, thus minimizing chronic morbidities and optimizing health-related quality of life [17]. As this app was developed through an in-depth pharmacokinetic study, it attempted to develop a family-centered approach while still understanding the intricate pharmacological aspects. However, as this was a feasibility study, pharmacokinetic models were not yet incorporated in detail.

Throughout the feasibility study, the need for a standardized and user-friendly method to interpret hearing results was identified. It was evident that doctors, audiologists, and nurses experienced difficulty in identifying a significant hearing loss and the need for dosage adjustments based on these audiologic results. Therefore, an ototoxicity monitoring protocol for DR-TB and mobile ototoxicity calculator app, *OtoCalc*, was developed. *OtoCalc* assists health care workers with the calculation of a significant hearing loss, its equivalent grade, and recommendations for management. In addition, it is specifically useful when audiologists may not be available to interpret data and can be used as an eHealth system.

This grading system is still in the premature phase and needs validation in an independent group of patients. This newly developed app needs to be trialed by various health care professionals such as doctors, nurses, pharmacists, and audiologists within the DR-TB population. It has the potential to standardize monitoring and dosage adjustments based on hearing while also allowing for reliable statistics and follow-up of the patients on a single database throughout the country.

### Limitations and Recommendations

Various aspects still need to be evaluated, such as costs (free Wi-Fi is not always available), user satisfaction, efficiency after implementation, and data security. A multisite study is required in at least two provinces in South Africa.

### Contribution to the Field

Despite the challenges and limitations that have been noted through this study and within the app itself, this app is the first of its kind and has the potential to make significant contributions to ototoxicity monitoring and management as well as the collection of more consistent and reliable statistics from uniform classification and measurement. The app provides a practical solution using existing infrastructure and technology (ie, smartphones) to assist various types of health care professionals dealing with patients with DR-TB to understand hearing loss and to assist with the appropriate management, specifically in cases where an audiologist is not present.

### Conclusions

*OtoCalc* is a potential tool to be used in monitoring ototoxicity in both resource-limited and resource-abundant countries. It has proven to be user friendly and time saving. *OtoCalc* has been tested in various settings and will continue being used as a vehicle to monitor ototoxicity. This technology could pave the way for future guidance and prevention of ototoxicity.

## Appendix

Multimedia Appendix 1 Mobile app grading system.

## Abbreviations

ASHA: American Speech-Language-Hearing Association
CTCAE: Common Terminology Criteria for Adverse Events
DR-TB: drug-resistant tuberculosis
eHealth: electronic health
mHealth: mobile health
TB: tuberculosis
TUNE: Theunissen and colleagues
