# Supplementary material for: An Ototoxicity Grading System Within a Mobile App (OtoCalc) for a Resource-Limited Setting to Guide Grading and Management of Drug-Induced Hearing Loss in Patients With Drug-Resistant Tuberculosis: Prospective, Cross-Sectional Case Series
Source: JMIR Mhealth Uhealth. 2020 Jan 14;8(1):e14036. doi: 10.2196/14036 (PMC6996762; doi:10.2196/14036)
Supplement: Multimedia Appendix 1 [file mhealth_v8i1e14036_app1.docx]

| **Grade** | **Description** | **Interpretation** | **Management recommendations** | **Recommendations for counselling** |
| --- | --- | --- | --- | --- |
| 1 | 10 dB or greater change in thresholds at two or more ultra-high frequencies (9 kHz to 20 kHz) from baseline in at least one ear | - The patient will likely not notice a deterioration of hearing abilities. - It is the early stages of hearing loss, but it is not yet affecting the frequency range of speech sounds (250Hz – 8 kHz). - It is possible that should the patient continue with the current drug regimen; the hearing loss may progress to speech frequencies. | *Goal:* Possible prevention or reduction of clinical hearing loss through early identification.  *Team:*  *Medical doctor:*   - Consider dosage adjustment. - Consider the use of an alternate non-ototoxic drug.   *Audiologist or nurse:*   - Inform the medical doctor of the change in thresholds. - Motivate for dosage adjustment. - Motivate for the use of an alternate non-ototoxic drug. - A follow-up appointment in 2 weeks for repeat audiogram. | *Goal:* Possible prevention or reduction of clinical hearing loss through early identification.  *Team:*  *Medical doctor:*   - Consider dosage adjustment. - Consider the use of an alternate non-ototoxic drug.   *Audiologist or nurse:*   - Inform the medical doctor of the change in thresholds. - Motivate for dosage adjustment. - Motivate for the use of an alternate non-ototoxic drug. - A follow-up appointment in 2 weeks for repeat audiogram. |
| 2 | 20 dB or greater change in thresholds at more than two frequencies in ultra-high range (9 kHz to 20 kHz) from baseline in at least one ear | - The patient will likely not notice a deterioration of hearing abilities unless he/she is a musician. - This is a significant loss of cochlear hair cell function in the ultra-high frequencies that are not yet affecting the frequency range of speech sounds (0.25 kHz – 8 kHz).   It is possible that should the patient continue with the current drug regimen; the hearing loss may progress to speech frequencies. | *Goal:* Possible prevention or reduction of clinical hearing loss through early identification.  *Team:*  *Medical doctor:*   - Consider dosage adjustment. - Consider the use of an alternate non-ototoxic drug.   *Audiologist or nurse:*   - Inform the medical doctor of the change in thresholds. - Motivate for dosage adjustment. - Motivate for the use of an alternate non-ototoxic drug. - A follow-up appointment in 2 weeks for repeat audiogram. | *Goal:* For patients to understand the nature and implications of hearing loss, but also the implications should they refuse treatment. It is important that patients feel supported and not alone.  *Team:*  *All health professionals should discuss the following with the patient and his/her family:*   - The possibility that some drugs used in the treatment of TB may cause a hearing loss. - Importance of adherence to treatment despite the potential hearing loss. - Hearing loss is not always preventable but that a motivation for the alternate non-ototoxic drug will be done. - Hearing loss will in most cases be permanent. - Hearing loss may impact on their communication abilities are home, work and with family or friends. - Amplification devices (e.g. hearing aids) are available if hearing loss is identified. - Discuss and address the fears and concerns of the patient and family members. - Provide the patient and his/her family with all the medical and audiological options relevant to the management of hearing loss.   The patient should report any change in medical and hearing status to health professionals. |
| 3 | 10 dB or greater change in thresholds into two frequencies from 8 kHz and below from baseline in at least one ear | - The hearing loss is now in the speech frequency range. - Depending on the patients' listening demands and processing abilities, the hearing loss may affect his/her day to day functioning. - The patient may hear people talking, however, may not always be able to discriminate the words spoken, for example ‘eight versus fate’. - The patient will likely also struggle to discriminate speech in the presence of background noise. | *Goal:* The identification of early clinical hearing loss, and exploration or alternative treatment to prevent/ reduce further hearing loss and functional impairment.  *Team:*  *Medical doctor:*   - Consider dosage adjustment - Consider the use of an alternate non-ototoxic drug. - Refer to audiologist for diagnostic audiological assessment.   *Nurse:*   - Inform the medical doctor of the change in thresholds. - Motivate for dosage adjustment. - Motivate for the use of an alternate non-ototoxic drug. - Refer to audiologist for diagnostic audiological assessment.   *Audiologist:*   - Conduct diagnostic audiological assessment. - Consider hearing amplification for future. - Inform the medical doctor of the change in thresholds. - Motivate for dosage adjustment. - Motivate for the use of an alternate non-ototoxic drug.   A follow-up appointment in 2 weeks for repeat audiogram. | *Goal:* For the patients to understand hearing loss, as well as its possible management in the future. It is important for patients to understand that DR-TB treatment is imperative, despite the hearing loss; but also, that they are not alone, and management is possible.  *Team:*  *All health professionals should discuss the following with the patient and his/her family:*   - The possibility that some drugs used in the treatment of TB may cause a hearing loss. - Importance of adherence to treatment despite the potential hearing loss. - Hearing loss is not always preventable but that a motivation for the alternate non-ototoxic drug will be done. - Hearing loss will in most cases be permanent. - Hearing loss may impact on their communication abilities are home, work and with family or friends. - The patient is not alone, and there are others who are also suffering from this hearing loss (try and put patients in touch with each other for support). - Amplification devices (e.g. hearing aids) are available if hearing loss is identified. - Discuss and address the fears and concerns of the patient and family members. - Provide the patient and his/her family with all the medical and audiological options relevant to the management of hearing loss.The patient should report any change in medical and hearing status to health professionals. |
| 4 | 10 dB or greater change in thresholds in any 3 or more frequencies from 8 kHz and below from baseline in at least one ear | - The hearing loss is now in the speech frequency range. - Depending on the patients' listening demands and processing abilities, the hearing loss may affect his/her day to day functioning. - The patient may hear people talking, however, may not always be able to discriminate the words spoken, for example ‘eight versus fate’. - The patient will likely also struggle to discriminate speech in the presence of background noise.   . | *Goal:* The identification of early clinical hearing loss, and exploration or alternative treatment to prevent/ reduce further hearing loss and functional impairment. Audiological management can be considered.  *Team:*  *Medical doctor:*   - Consider dosage adjustment. - Consider the use of an alternate non-ototoxic drug. - Refer to audiologist for diagnostic audiological assessment.   *Nurse:*   - Inform the medical doctor of the change in thresholds. - Motivate for dosage adjustment. - Motivate for the use of an alternate non-ototoxic drug. - Refer to audiologist for diagnostic audiological assessment.   *Audiologist:*   - Conduct diagnostic audiological assessment. - Consider hearing amplification. - Discuss communication strategies with the patient and his/her family. - Inform the medical doctor of the change in thresholds. - Motivate for dosage adjustment. - Motivate for the use of an alternate non-ototoxic drug.   A follow-up appointment in 2 weeks for repeat audiogram. | **Goal:** For the patients to understand hearing loss, as well as its possible management in the future. It is essential for patients to understand that DR-TB treatment is imperative, despite the hearing loss; but also, that they are not alone, and management is possible. Patients need to start understanding the process of amplification.  *Team:*  *All health professionals should discuss the following with the patient and his/her family:*   - The possibility that some drugs used in the treatment of TB may cause a hearing loss. - Importance of adherence to treatment despite the potential hearing loss. - Hearing loss is not always preventable but that a motivation for the alternate non-ototoxic drug will be done. - Hearing loss will in most cases be permanent. - Hearing loss may impact on their communication abilities are home, work and with family or friends. - The patient is not alone, and there are others who are also suffering from this hearing loss (try and put patients in touch with each other for support). - Amplification devices (e.g. hearing aids) are available. - Discuss and address the fears and concerns of the patient and family members. - Provide the patient and his/her family with all the medical and audiological options relevant to the management of hearing loss. - The patient should report any change in medical and hearing status to health professionals. |
| 5 | 20 dB and above change in thresholds at 3 frequencies or more at 8 kHz or below from baseline in at least one ear | - This hearing loss is affecting the patients' ability to hear, discriminate and understand speech. - The hearing loss is likely to affect the patient considerably in communicating at home, work and with family or friends. - The patient will struggle significantly to hear speech in noisy situations. - The hearing difficulties may affect the patient socially and emotionally. | *Goal:* The prevention or reduction of further hearing loss by treatment adjustments/changes. The audiological team can consider amplification.  *Team:*  *Medical doctor:*   - Consider dosage adjustment. - Consider the use of an alternate non-ototoxic drug. - Refer to audiologist for diagnostic audiological assessment.   *Nurse:*   - Inform the medical doctor of the change in thresholds. - Motivate for dosage adjustment. - Motivate for the use of an alternate non-ototoxic drug. - Refer to audiologist for diagnostic audiological assessment.   *Audiologist:*   - Conduct diagnostic audiological assessment. - Consider hearing amplification. - Discuss communication strategies with the patient and his/her family. - Inform the medical doctor of the change in thresholds. - Motivate for dosage adjustment. - Motivate for the use of an alternate non-ototoxic drug.   A follow-up appointment in 2 weeks for repeat audiogram. | *Goal:* For the patients to understand hearing loss, as well as its possible management in the future. It is important for patients to understand that DR-TB treatment is imperative, despite the hearing loss; but also, that they are not alone, and management is possible. Patients need to start understanding the process of amplification.  *Team:*  *All health professionals should discuss the following with the patient and his/her family***:**   - The possibility that some drugs used in the treatment of TB may cause a hearing loss. - Importance of adherence to treatment despite the potential hearing loss. - Hearing loss is not always preventable but that a motivation for the alternate non-ototoxic drug will be done. - Hearing loss will in most cases be permanent. - Hearing loss may impact on their communication abilities are home, work and with family or friends. - The patient is not alone, and there are others who are also suffering from this hearing loss (try and put patients in touch with each other for support). - Amplification devices (e.g. hearing aids) are available. - Discuss and address the fears and concerns of the patient and family members. - Provide the patient and his/her family with all the medical and audiological options relevant to the management of hearing loss. The patient should report any change in medical and hearing status to health professionals. |
| 6 | More than 40 dB change in threshold in up to 2 frequencies from 8 kHz and below from baseline in both ears | - This hearing loss is affecting the patients' ability to hear, discriminate and understand speech. - The hearing loss is likely to affect the patient considerably in communicating at home, work and with family or friends. - The patient will struggle significantly to hear speech in noisy situations. - The hearing difficulties may affect the patient socially and emotionally. | **Goal:** Treatment changes are imperative. And audiological management is essential at this stage, if possible.  *Team:*  *Medical doctor:*   - Consider dosage adjustment. - Consider the use of an alternate non-ototoxic drug. - Refer to audiologist for diagnostic audiological assessment.   *Nurse:*   - Inform the medical doctor of the change in thresholds. - Motivate for dosage adjustment. - Motivate for the use of an alternate non-ototoxic drug. - Refer to audiologist for diagnostic audiological assessment.   *Audiologist:*   - Conduct diagnostic audiological assessment. - Consider hearing amplification. - Discuss communication strategies with the patient and his/her family. - Inform the medical doctor of the change in thresholds. - Motivate for dosage adjustment. - Motivate for the use of an alternate non-ototoxic drug.   A follow-up appointment in 2 weeks for repeat audiogram. | **Goal:** The patient must understand that if he/she stops treatment, the hearing will not recover, and it will have life-threatening implications. The patient must start coming to terms with the possibility of losing their hearing entirely, and its life-changing implications ahead.  *Team:*  *All health professionals should discuss the following with the patient and his/her family:*   - The possibility that some drugs used in the treatment of TB may cause a hearing loss. - Importance of adherence to treatment despite the potential hearing loss. - Hearing loss is not always preventable but that a motivation for the alternate non-ototoxic drug will be done. - Hearing loss will in most cases be permanent. - Hearing loss may impact on their communication abilities are home, work and with family or friends. - The patient is not alone, and there are others who are also suffering from this hearing loss (try and put patients in touch with each other for support). - Amplification devices (e.g. hearing aids) are available. - Discuss and address the fears and concerns of the patient and family members. - Provide the patient and his/her family with all the medical and audiological options relevant to the management of hearing loss.   The patient should report any change in medical and hearing status to health professionals. |
| 7 | More than 40 dB change in thresholds at 3 or more frequencies from 8 kHz and below from baseline both ears | - This hearing loss is affecting the patients' ability to hear, discriminate and understand speech. - The hearing loss is likely to affect the patient considerably in communicating at home, work and with family or friends. - The patient will struggle significantly to hear speech in noisy situations. - The patient will struggle significantly to hear in small group situations. - The patient will struggle significantly with one-to-one conversation. - The hearing difficulties may affect the patient socially and emotionally. | *Goal:* Treatment changes are imperative. And audiological management is essential at this stage, if possible.  *Team:*  *Medical doctor:*   - Consider dosage adjustment. - Consider the use of an alternate non-ototoxic drug. - Refer to audiologist for diagnostic audiological assessment.   *Nurse:*   - Inform the medical doctor of the change in thresholds. - Motivate for dosage adjustment. - Motivate for the use of an alternate non-ototoxic drug. - Refer to audiologist for diagnostic audiological assessment.   *Audiologist:*   - Conduct diagnostic audiological assessment. - Consider hearing amplification. - Discuss communication strategies with the patient and his/her family. - Inform the medical doctor of the change in thresholds. - Motivate for dosage adjustment. - Motivate for the use of an alternate non-ototoxic drug. - A follow-up appointment in 2 weeks for repeat audiogram. | *Goal:* The patient must understand that if he/she stops treatment, the hearing will not recover, and it will have life-threatening implications. The patient must start coming to terms with the possibility of losing their hearing altogether, and its life-changing implications ahead.  *Team:*  *All health professionals should discuss the following with the patient and his/her family:*   - The possibility that some drugs used in the treatment of TB may cause a hearing loss. - Importance of adherence to treatment despite the potential hearing loss. - Hearing loss is not always preventable but that a motivation for the alternate non-ototoxic drug will be done. - Hearing loss will in most cases be permanent. - Hearing loss may impact on their communication abilities are home, work and with family or friends. - The patient is not alone, and there are others who are also suffering from this hearing loss (try and put patients in touch with each other for support). - Amplification devices (e.g. hearing aids) are available. - Discuss and address the fears and concerns of the patient and family members. - Provide the patient and his/her family with all the medical and audiological options relevant to the management of hearing loss. The patient should report any change in medical and hearing status to health professionals. |
| 8 | Hearing thresholds at 80 dB or greater in 4 or more speech frequencies (250 Hz to 8 kHz) in both ears | - The patient has now lost most of his/her hearing. - The patient will most likely be unable to communicate with family, friends and colleagues effectively. - The patient will, as a result, be isolated in various ways, specifically in social and occupational situations. - Depending on the patient’s job, it is possible that he/she will be unable to continue work due to the hearing disability. | *Goal:* Cochlear implant consideration and referral audiological considerations for long-term management.  *Team:*  *Medical doctor and nurse:*   - Refer to an audiologist for aural rehabilitation. | *Goal:* This is an extremely vulnerable place for patients, as they are often entirely isolated. It is important they understand that they are not alone and that there are still options for jobs and life in the Deaf world. Facilitation into this ‘Deaf’ world needs to be guided, addressing all the fears and concerns.  *Team:*  *All health professionals should discuss the following with the patient and his/her family:*   - The possibility that some drugs used in the treatment of TB may cause a hearing loss. - Importance of adherence to treatment despite the potential hearing loss. - Hearing loss is not always preventable but that a motivation for the alternate non-ototoxic drug will be done. - Hearing loss will in most cases be permanent. - Hearing loss may impact on their communication abilities are home, work and with family or friends. - The patient is not alone, and there are others who are also suffering from this hearing loss (try and put patients in touch with each other for support). - Amplification devices (e.g. hearing aids) are available. - Discuss and address the fears and concerns of the patient and family members. - Provide the patient and his/her family with all the medical and audiological options relevant to the management of hearing loss.   The patient should report any change in medical and hearing status to health professionals. |
